# Back to the Basics: Resting State Functional Connectivity of the Reticular Activation System in PTSD and its Dissociative Subtype

**DOI:** 10.1177/2470547019873663

**Published:** 2019-09-27

**Authors:** Janine Thome, Maria Densmore, Georgia Koppe, Braeden Terpou, Jean Théberge, Margaret C. McKinnon, Ruth A. Lanius

**Affiliations:** 1 Department of Psychiatry, Western University, London, Ontario, Canada; 2 Department of Theoretical Neuroscience, Central Institute of Mental Health Mannheim, Medical Faculty Mannheim, Heidelberg University, Mannheim, Germany; 3 Department of Psychiatry, Central Institute of Mental Health Mannheim, Medical Faculty Mannheim, Heidelberg University, Mannheim, Germany; 4 Imaging Division, Lawson Health Research Institute, London, Ontario, Canada; 5 Department of Neuroscience, Western University, London, Ontario, Canada; 6 Department of Medical Biophysics, Western University, London, Ontario, Canada; 7 Homewood Research Institute, Guelph, Ontario, Canada; 8 Mood Disorder Programs, St. Joseph's Healthcare, Hamilton, Ontario, Canada; 9 Department of Psychiatry and Behavioral Neurosciences, McMaster University, Hamilton, Ontario, Canada

**Keywords:** post-traumatic stress disorder, dissociation, resting state functional magnetic resonance imaging, reticular activation system, pedunculopontine nuclei

## Abstract

**Background:**

Brainstem and midbrain neuronal circuits that control innate, reflexive responses and arousal are increasingly recognized as central to the neurobiological framework of post-traumatic stress disorder (PTSD). The reticular activation system represents a fundamental neuronal circuit that plays a critical role not only in generating arousal but also in coordinating innate, reflexive responding. Accordingly, the present investigation aims to characterize the resting state functional connectivity of the reticular activation system in PTSD and its dissociative subtype.

**Methods:**

We investigated patterns of resting state functional connectivity of a central node of the reticular activation system, namely, the pedunculopontine nuclei, among individuals with PTSD (n = 77), its dissociative subtype (PTSD+DS; n = 48), and healthy controls (n = 51).

**Results:**

Participants with PTSD and PTSD+DS were characterized by within-group pedunculopontine nuclei resting state functional connectivity to brain regions involved in innate threat processing and arousal modulation (i.e., midbrain, amygdala, ventromedial prefrontal cortex). Critically, this pattern was most pronounced in individuals with PTSD+DS, as compared to both control and PTSD groups. As compared to participants with PTSD and controls, individuals with PTSD+DS showed enhanced pedunculopontine nuclei resting state functional connectivity to the amygdala and the parahippocampal gyrus as well as to the anterior cingulate and the ventromedial prefrontal cortex. No group differences emerged between PTSD and control groups. In individuals with PTSD+DS, state derealization/depersonalization was associated with reduced resting state functional connectivity between the left pedunculopontine nuclei and the anterior nucleus of the thalamus. Altered connectivity in these regions may restrict the thalamo-cortical transmission necessary to integrate internal and external signals at a cortical level and underlie, in part, experiences of depersonalization and derealization.

**Conclusions:**

The present findings extend the current neurobiological model of PTSD and provide emerging evidence for the need to incorporate brainstem structures, including the reticular activation system, into current conceptualizations of PTSD and its dissociative subtype.

## Introduction

The spontaneous neuronal activation observed during resting state functional magnetic resonance imaging (fMRI) is frequently utilized to illustrate psychopathological alterations in cortical and in subcortical brain networks in psychiatric disorders, serving as a key biomarker in post-traumatic stress disorder (PTSD).^1–14^ Importantly, emerging evidence has underscored the importance of incorporating deep-layer midbrain/brainstem neural circuits into the neurobiological framework of PTSD.^1,2,15–19^ The reticular activation system (RAS) serves a fundamental role toward the gating of salient, environmental information to higher order, cortical brain structures to facilitate the generation and the maintenance of an arousal state.^20–26^ Moreover, the RAS has also been linked to support the formation of action–outcome associations in the brain.^20–26^ Hence, the RAS plays a crucial role in supporting reflexive processing, as it shapes the general arousal state of the organism and provides the foundation for innate, defensive responding.^26^ Critically, however, prolonged and repeated traumatic experiences may lead to permanent alterations in these fundamental neuronal circuitries.^14,18,27–32^

An important function of the RAS is to promote an arousal state throughout the brain, necessary particularly in the face of immediate danger. The RAS transmits salient information to numerous subcortical and cortical structures, mainly, but not exclusively, via ascending projections through thalamic nuclei (e.g., anterior, medial dorsal, pulvinar nuclei), leading to its identification as “the gatekeeper to consciousness”.^33,37^ As a result, it has been proposed that the RAS serves a critical role in transitioning between brain states ranging from lower conscious sleep states to states of wakeful presence.^21,38–41^ These findings aid greatly in our understanding of PTSD, where PTSD is characterized by frequent shifts in states of arousal, ranging from hyperarousal states, which are more often associated with hypervigilance symptoms, to hypoarousal states, which are more often related to dissociative symptoms (i.e., emotional numbing, depersonalization, derealization); the latter pattern of symptoms is predominant in the recently formulated dissociative subtype.^13,14^ Whereas a pattern of decreased brain activation in prefrontal emotion regulatory regions (e.g., ventromedial prefrontal cortex) and increased activation in emotion generating regions (e.g., periaqueductal gray and amygdala) has been associated with hyperarousal, the opposite pattern of neural activation is indicative of emotional detachment in participants with PTSD and its dissociative subtype, respectively. Notably, this pattern is observed during conditions of both symptom provocation (e.g., traumatic script) and during resting state.^1,2,13–15,17,42–45^

Post-traumatic stress disorder has been further associated with altered threat-related processing, demonstrated by alterations in reaction time, physiological responding (e.g., startle response, heart rate), and the recruitment of brain regions involved in emotion processing (e.g., periaqueductal gray, amygdala) and stimulus evaluation (e.g., inferior orbitofrontal cortex, anterior cingulate cortex) to threat- or trauma-related cues.^5,46–56^ Importantly, recent studies emphasize sensitization, particularly in innate threat processing-related brain regions in PTSD, where the presentation of subliminal threat cues elicited stronger activation in the brainstem, the midbrain, the amygdala, and the parahippocampal gyrus in individuals with PTSD as compared to healthy trauma- and non-trauma-exposed controls.^5,57–63^

Despite emerging evidence of altered states of arousal, and the sensitization of innate threat processing in PTSD during symptom provocation and resting state,^1,2,5,43,44,57–62,64–67^ research examining connectivity of the RAS to subcortical and to cortical brain structures remains in its nascent stages. Accordingly, we sought to delineate resting state functional connectivity (rsFC) patterns of a main component of the RAS, the pedunculopontine nuclei (PPN),^39,68–75^ among individuals with PTSD, its dissociative subtype (PTSD+DS), and healthy controls.

We hypothesized that as compared to controls, both PTSD groups would show altered PPN rsFC to brain regions involved in innate threat processing and arousal (e.g., midbrain, amygdala). Moreover, we hypothesized that individuals with PTSD+DS and PTSD would differ in their PPN rsFC patterns to cortical brain regions involved in emotion regulation (e.g., ventromedial prefrontal cortex).

## Methods

### Sample Description

The present investigation included 176 participants: 125 participants met the criteria for PTSD and 51 participants were free of any mental disorder throughout their life (control group). Of the 125 participants meeting criteria for PTSD, 77 individuals met criteria for PTSD without the dissociative subtype and the remaining 48 individuals met criteria for the dissociative subtype of PTSD (PTSD+DS). Details on exclusion criteria can be found in Supplemental Information S1.

Post-traumatic stress disorder diagnoses and symptom severity were assessed using the Clinician-Administered PTSD Scale (CAPS 4,CAPS 5).^76^ Comorbid Axis I disorders were diagnosed with the Structured Clinical Interview for DSM-IV Axis I Disorders (SCID-I).^77^ Both measures were administered by a trained clinical psychologist.

Childhood traumatization was assessed by the Childhood Trauma Questionnaire (CTQ).^78^ The severity of depressive symptomatology and trait dissociation were assessed with the Beck Depression Inventory (BDI)^79^ and the Multiscale Dissociation Inventory (MDI),^80^ respectively. Immediately after the scanning session was complete, state anxiety (three items of the State-Trait Anxiety Inventory (STAI));^81^ and state derealization/depersonalization inventories were administered (Response to Script-Driven Imagery Scale; RSDI). See Supplemental Information S1 for details on statistical analyses.^82^

Scanning took place either at the Robarts Research Institute's Centre for Functional and Metabolic Mapping or the Lawson Health Research Institute for Imaging in London, Ontario, Canada. The study was approved by the research ethics board at Western University of Canada, and all subjects provided written informed consent.

### Resting State fMRI Data Acquisition

All fMRI images were collected using a 3.0 T whole-body MRI scanner (Magnetom Tim Trio, Siemens Medical Solutions, Erlangen, Germany) with a manufacturer's 32-channel phased array head coil. T1-weighted anatomical images were collected with 1 mm isotropic resolution [MP-RAGE, TR/TE/TI = 2300 ms/2.98 ms/900 ms, FA 9 º, FOV = 256 mm × 240 mm × 192 mm, acceleration factor = 4, total acquisition time = 192 s; (FOV = field of view; TR = time resolution; TE=echotime; FA = flip angle)].

Blood-oxygenation level-dependent signal (BOLD) fMRI images were obtained with the standard gradient-echo planar imaging (EPI) pulse sequence. EPI volumes were acquired with 2 mm isotropic resolution (FOV = 192 mm × 192 mm × 128 mm (94 × 94 matrix, 64 slices), TR/TE = 3000 ms/20 ms, flip angle = 90 °, 120 volumes).

Participants were instructed to close their eyes and let their minds wander during the 6-min resting scan.

### fMRI Data Preprocessing

Image preprocessing and statistical analyses were conducted using Statistical Parametric Mapping (SPM12, Wellcome Trust Center of Neuroimaging, London, UK; http://www.fil.ion.ucl.ac.uk/spm) and the spatially unbiased infratentorial template (SUIT) toolbox (version, 3.1)^83^ implemented in Matlab R2018b (MathWorks).

The location of the origin of the anatomical images was checked and, in cases of deviation, manually set to the anterior commissure. Functional images were reoriented based on their anatomical image. The 120 (reoriented) functional images were realigned to the first image and resliced to the mean functional image. In addition, six realignment parameters for changes in motion across the different planes were derived. To ensure motion correction, we used the Artifact Detection Tool (ART) software package^84^ (at 2 mm motion threshold; ART software; Gabrieli Lab; McGovern Institute for Brain Research, Cambridge, MA; www.nitrc.org/projects/artifact_detect)^84^ to compute regressors accounting for motion outlier volumes that were in addition to the six movement regressors computed during standard realignment.

#### fMRI Data Preprocessing: Brainstem and Cerebellum

To improve the voxel-by-voxel normalization of the midbrain, lower brainstem, and cerebellum and hence, to enhance the depiction and signal extraction of the PPN, functional and anatomical data were normalized to the SUIT template (version 3.1)^83,85^ by applying the following steps: (1) whole-brain anatomical images were first segmented and then cropped, retaining only the cerebellum and brainstem; (2) the partial-brain anatomical images were normalized using the SUIT-normalize function that creates a nonlinear deformation map to the SUIT template by applying the cosine-basis approach introduced by Ashburner; (3) the realigned and resliced functional images (see “fMRI Data Preprocessing: Brainstem and Cerebellum” section) were normalized by applying the deformation matrix generated in step 2, cropped (retaining the cerebellum and brainstem only, i.e., functional partial-brain) and resliced to a voxel size of 1.5 × 1.5 × 1.5 mm^3^; (4) partial-brain functional data were smoothed with a Gaussian filter of 4 mm full-width at half-maximum (FWHM) and band-pass filtered with a high-pass filter of .01 Hz and a low-pass filter of .08 Hz.^86,87^

#### fMRI Data Preprocessing: Whole Brain

The realigned and resliced functional images (see “fMRI Data Preprocessing” section) were coregistered to the anatomical image for each subject. Coregistration was followed by the segmentation of the images into each tissue type (gray and white matter as well as cerebrospinal fluid), spatial normalization to the Montreal Neurological Institute (MNI) standard template, smoothing with a 6 mm FWHM Gaussian kernel, and band-pass filtering with a high-pass filter of .01 Hz and low-pass filter of .08 Hz.^86,87^

### rsFC Analyses

#### Seed Region Definition

Seed masks for the right and the left PPN were generated using the WFU PickAtlas software (Functional MRI Laboratory, Wake Forest University School of Medicine)^88^ by defining 4 mm spheres around the following coordinates: x = ±7, y = −32, z = −22.^89^ The seed region was then confirmed visually using Duvernoy's Atlas.^90^ Using self-written MATLAB scripts, the mean signal BOLD time course of each seed (i.e., the right and left PPN) was extracted from the partial-brain data, ensuring enhanced spatial accuracy of the defined seed regions (see “fMRI Data Preprocessing: Brainstem and Cerebellum” section).

#### First Level

For each seed, separate voxel-wise first-level multiple regression models were set up, including the seed time course (i.e., regressor of interest), as well as the ART regressor indicating motion outliers and realignment parameters (i.e., regressors of no interest). Regression analyses were performed at the whole-brain level and separately for the brainstem and the cerebellum, as normalization to the SUIT template allows for increased spatial accuracy at the brainstem/cerebellum level (i.e., partial-brain level).

#### Second Level: Within-Group Analyses

**Figure 1. fig1-2470547019873663:**
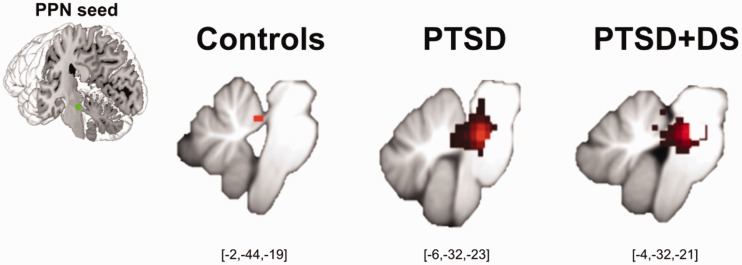
Partial-brain resting state functional connectivity (rsFC) of the left PPN within controls, PTSD, and PTSD+DS separately. RsFC at a local significance threshold of *p* < .05, FWE corrected, partial-brain level (i.e., SUIT space) displayed for each group separately (red color). The seed region (PPN) is displayed in green. Controls exhibited rsFC of the left PPN (within group) with the left cerebellar anterior lobule I to IV only. PTSD exhibited rsFC of the left PPN (within group) with a cluster encompassing the PPN itself, the locus coeruleus, the superior colliculi, the midbrain reticular formation, the left vermis IV to V, and the right cerebellar posterior lobule VI. PTSD+DS exhibited rsFC of the left PPN (within group) with the PPN itself, the locus coeruleus, and the right cerebellar anterior lobule I to IV (see also Tables S1 and S2). PPN: pedunculopontine nuclei; PTSD: post-traumatic stress disorder; PTSD+DS: dissociative subtype of PTSD.

To explore rsFC patterns of the seed within groups, separate one-sample T-Tests (i.e., right PPN, left PPN) were conducted voxel-wise with regard to rsFC at the whole-brain level (see also Tables S4 and S5) as well as at the partial-brain level (Figure 1, Supplemental Information S1; Tables S1 and S2).

#### Second Level: Between-Group Analyses

To compare rsFC patterns of the PPN between groups, we utilized a flexible-factorial design with the factor group (controls vs. PTSD vs. PTSD+DS) and the factor hemisphere (left PPN vs. right PPN) to test for a significant group × hemisphere interaction with regard to rsFC at the whole-brain level (see also Table S6) and at the partial-brain level (i.e., brainstem/ cerebellum). The latter is included in Supplemental Information only (Table S3).

#### Second Level: rsFC and Clinical Symptomatology

Multiple regression analyses were conducted to explore the association between rsFC of the seed regions (i.e., right and left PPN) and clinical variables, including PTSD symptom severity (CAPS), childhood traumatization (CTQ), depressive symptomatology (BDI), and state depersonalization/derealization (RSDI derealization/depersonalization).

#### Analyses Approach and Statistical Thresholding

Resting state functional connectivity was analyzed using a region-of-interest (ROI) approach, with a priori brain regions, namely, the midbrain, the amygdala, and the ventromedial prefrontal cortex, selected due to their relation to innate threat processing and arousal.^18,19,57,60–63,91–93^ We also included the thalamus, as it serves as a major hub in transmitting information from the RAS to cortical and to subcortical structures. Bilateral amygdala, thalamus, and ventromedial prefrontal masks were created with the automated anatomical labeling atlas,^94^ which was implemented in the WFU PickAtlas software.^88^ A midbrain mask was adopted from the Harvard Ascending Arousal Network (AAN) atlas.^23^

All ROI results were reported at a local significance threshold of *p* < .05 (voxel-level), with an alpha-level adjustment for multiple comparisons (family-wise error (FWE) correction). In addition, a Bonferroni adjustment was applied according to the number of tested ROIs (N = 4), leading to a local significance threshold of *p* < .0125, FWE corrected. Whole-brain results for group differences with a local significance threshold of *p* < .001, *k* > 10, uncorrected for multiple comparisons can be found in the Supplemental Information only (Table S6).

## Results

### Sociodemographic and Clinical Information

Although groups did not differ in age or gender, significant group differences emerged for all clinical and subjective experience measurements (see Table 1 for details).

**Table 1.** Demographic and clinical characteristics of the study sample.

				Test- statistic	*p*	Post-Hoc Comparison								
						Controls vs. PTSD			Controls vs. PTSD+DS			PTSD vs. PTSD+DS		
	Controls N = 51	PTSD N = 77	PTSD+DS N = 48			Test- statistic	df	*p*	Test- statistic	df	*p*	Test- statistic	df	*p*
*Demographics*														
Gender (n)														
Female	34	47	39	5.29^a^	.071	–	–	–	–	–	–	–	–	–
Male	17	30	9											
Age mean (SD)	35.02 (11.55)	38.82 (11.91)	40.02 (13.59)	2.77^b^	.066	–	–	–	–	–	–	–	–	–
*Clinical characteristics*														
CAPS total mean (SD)	0.63 (2.72)	56.51 (19.67)	64.09 (22.99)	191.97^b^	<.001*	20.19^c^	125	<.001*	19.57^c^	92	<.001*	1.89^c^	117	.060
MDI				105.88^b^	<.001*									
MDI total mean (SD)	34.04 (3.99)	54.29 (15.09)	81.53 (22.37)			9.25^c^	120	<.001*	14.74^c^	88	<.001*	7.67^c^	110	<.001*
MDI derea mean (SD)	5.22 (0.62)	8.65 (3.28)	13.50 (4.43)			7.31^c^	120	<.001*	13.09^c^	88	<.001*	6.59^c^	110	<.001*
MDI deperso mean (SD)	5.20 (0.64)	6.76 (2.71)	13.05 (5.79)			4.00^c^	120	<.001*	9.52^c^	88	<.001*	7.81^c^	110	<.001*
*Trauma history*														
CTQ total mean (SD)	32.35 (9.18)	56.82 (22.97)	69.22 (19.64)	46.76^b^	<.001*	7.08^c^	119	<.001*	11.79^c^	91	<.001*	2.98^c^	114	.004*
				5.98^b^	<.001*									
Emotional abuse mean (SD)	6.88 (2.79)	13.94 (6.86)	17.02 (5.49)			6.83^c^	119	<.001*	11.39^c^	91	<.001*	2.52^c^	114	.013
Physical abuse mean (SD)	5.61 (1.55)	9.62 (4.97)	10.23 (4.01)			5.47^c^	119	<.001*	6.25^c^	91	<.001*	0.64^c^	114	.526
Sexual abuse mean (SD)	5.47 (2.12)	9.76 (6.33)	14.02 (7.73)			4.58^c^	119	<.001*	7.44^c^	91	<.001*	3.23^c^	114	.002*
Emotional neglect mean (SD)	8.31 (3.67)	14.24 (6.14)	16.73 (5.08)			6.06^c^	119	<.001*	9.23^c^	91	<.001*	2.26^c^	114	.026
Physical neglect mean (SD)	6.08 (2.14)	9.25 (4.34)	11.23 (4.52)			4.73^c^	119	<.001*	7.13^c^	91	<.001*	2.34^c^	114	.021
Combat exposure^d^				2.63^a^	.269									
Yes (N)	3	13	6			–	–	–	–	–	–	–	–	–
No (N)	42	64	42			–	–	–	–	–	–	–	–	–
*State characteristics*														
RSDI disso mean (SD)	2.74 (0.47)	3.69 (1.36)	4.98 (2.02)	25.69^b^	<.001*	4.66^c^	109	<.001*	7.44^c^	74	<.001*	3.52^c^	87	.001*
STAI-S mean (SD)	3.55 (1.17)	5.75 (2.15)	6.33 (2.43)	28.20^b^	<.001*	6.47^c^	109	<.001*	6.73^c^	74	<.001*	1.16^c^	87	.298

Note: CAPS: Clinician-Administered PTSD Scale; CTQ: Childhood Trauma Questionnaire; Derea: Derealization; Deperso: Depersonalization; MDI: Multiscale Dissociation Inventory; RSDI Disso: Dissociation Items from the Response to Script-Driven Imagery Scale; STAI-S: State-Trait Anxiety Inventory-State part; PTSD: post-traumatic stress disorder, PTSD+DS: PTSD with dissociative subtype; *p: p*-value.

a Kruskall–Wallis test.

b F test.

c T Test.

d Information is missing for six healthy control individuals.

* Significant at a threshold of *p* < 0.05, or in case of a significant effect, *p*-value is Bonferroni-adjusted according to the number of post-hoc comparisons.

### PPN Within-Group rsFC

#### Controls: Left PPN

Controls did not show significant rsFC of the left PPN to any other brain region.

#### Controls: Right PPN

**Figure 2.**
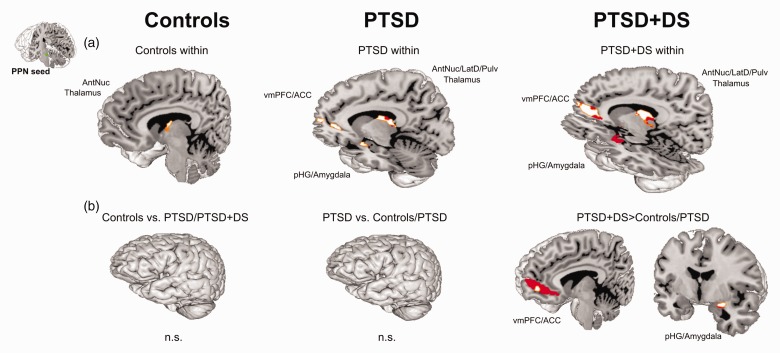
Resting state functional connectivity (rsFC) of the left and right PPN within controls, PTSD, and PTSD+DS separately (a) as well as group comparisons of the left and right PPN (b). ROI approach: rsFC results are reported at a local significance threshold of *p* < .0125, FWE corrected (additionally alpha-level adjustment according to the number of ROIs); only significant voxels surviving the latter threshold are displayed (binary, MNI space). RsFC is displayed in red (a: darker red = left PPN, lighter red = right PPN; b: darker red = PTSD+DS > controls; lighter red = PTSD+DS > PTSD). ACC: anterior cingulate cortex; AntNuc: anterior thalamic nucleus; n.s.: not significant; pHG: parahippocampal gyrus; PPN: pedunculopontine nuclei; PTSD: post-traumatic stress disorder; PTSD+DS: dissociative subtype of PTSD; vmPFC: ventromedial prefrontal cortex.

Controls showed significant rsFC of the right PPN and the right anterior nucleus of the thalamus (*p*_FWE_ = .001) (Figure 2(a)).

#### PTSD: Left PPN

Individuals with PTSD showed significant rsFC of the left PPN with a cluster encompassing the bilateral anterior, lateral dorsal, and pulvinar nuclei of the thalamus (all *p*_FWE_ < .011), and a cluster encompassing the right anterior cingulate cortex and the ventromedial prefrontal cortex (*p*_FWE_ = .004) (Figure 2(a)).

#### PTSD: Right PPN

Individuals with PTSD exhibited significant rsFC of the right PPN with a cluster encompassing the bilateral anterior, medial dorsal, lateral dorsal, and pulvinar nuclei of the thalamus (all *p*_FWE_ < .001), a cluster encompassing the left amygdala and the parahippocampal gyrus (*p*_FWE_ < .001), and a cluster encompassing the right anterior cingulate cortex and the ventromedial prefrontal cortex (*p*_FWE_ = .005) (Figure 2(a)).

#### PTSD+DS: Left PPN

Individuals with PTSD+DS revealed rsFC of the left PPN with a cluster encompassing the right anterior, midline, medial dorsal, and lateral dorsal nuclei of the thalamus (*p*_FWE_ = .009), a cluster encompassing the bilateral amygdala and the parahippocampal gyri (all *p*_FWE_ < .003), and a cluster encompassing the right anterior cingulate cortex and the ventromedial prefrontal (all *p*_FWE_ < .009) (Figure 2(a)).

#### PTSD+DS: Right PPN

Individuals with PTSD+DS were characterized by significant rsFC of the right PPN with a cluster encompassing the right anterior, lateral dorsal, and pulvinar nuclei of the thalamus (*p*_FWE_ = .006), a cluster encompassing the left medial dorsal and pulvinar nuclei of the thalamus (*p*_FWE_ = .009), a cluster encompassing the bilateral amygdala and the parahippocampal gyri (all *p*_FWE_ < .009), and a cluster encompassing the left anterior cingulate cortex and the ventromedial prefrontal cortex (*p*_FWE_ = .012) (Figure 2(a)).

### PPN Between-Group rsFC

The flexible-factorial analysis of variance showed a main effect of group (*p*_FWE_ < .018; alpha-level adjustment with FWE correction, without additional Bonferroni correction). Groups differed in rsFC of the PPN with a cluster encompassing the right amygdala and the parahippocampal gyrus (*p*_FWE_ = .014), and a cluster encompassing the left anterior cingulate cortex and the ventromedial prefrontal cortex (*p*_FWE_ = .018). We did not observe a main effect of hemisphere nor an interaction between the factors group and hemisphere.

#### Controls Versus PTSD

We did not observe significantly increased rsFC of the PPN with any other brain regions when comparing controls to individuals with PTSD (i.e., controls > PTSD; PTSD > controls).

#### Controls Versus PTSD+DS

We did not observe significantly stronger rsFC of the PPN with any other brain regions in controls as compared to individuals with PTSD+DS.

Individuals with PTSD+DS as compared to controls exhibited significantly stronger rsFC of the PPN with a cluster encompassing the right amygdala and the parahippocampal gyrus (*p*_FWE_ = .010), and a cluster encompassing the left anterior cingulate and the ventromedial prefrontal cortex (*p*_FWE_ = .002) (Table 2; Figure 2(b)).

**Table 2.** Between-group comparisons of resting state functional connectivity of the left and the right pedunculopontine nuclei.

	L/R	Brain region		k	Z	*p*_FWEcorr_	*p*_uncorr_	Peak MNI coordinate		
								x	y	z
Main effect of group										
	R	Amygdala/parahippocampal gyrus	*	68	3.44	.014	<.001	28	−4	−14
	L	Ventromedial prefrontal/anterior cingulate cortex	*	404	3.61	.018	<.001	−2	50	−12
Main effect of hemisphere										
			n.s.							
Interaction group × hemisphere										
			n.s.							
Between-group comparison										
Controls > PTSD			n.s.							
Controls > PTSD+DS			n.s.							
PTSD > controls			n.s.							
PTSD > PTSD+DS			n.s.							
PTSD+DS > controls	L	Ventromedial prefrontal/anterior cingulate cortex	**	405	4.15	.002	<.001	−2	50	−12
	R	Amygdala/parahippocampal gyrus	**	46	3.50	.010	<.001	28	−4	−14
PTSD+DS > PTSD	L	Ventromedial prefrontal/anterior cingulate cortex	**	172	3.71	.011	<.001	−12	54	−2
	R	Amygdala/parahippocampal gyrus	**	54	3.71	.005	<.001	28	−6	−14

Note: ROI approach: rsFC results are reported at a local significance threshold of *p < .05, FWE corrected and **p < .0125 (i.e., adjusted for number of ROIs), FWE corrected. PTSD: post-traumatic stress disorder; PTSD+DS: PTSD with the dissociative subtype; L: left; R: right; n.s. = no significant difference; k: cluster size; *p*_uncorr_: *p*-value, uncorrected for multiple comparisons; *p*_FWEcorr_: *p*-value, corrected for multiple comparisons (FWE); FWE: family-wise error; MNI: Montreal Neurological Institute.

#### PTSD Versus PTSD+DS

We did not observe significantly stronger rsFC of the PPN with any other brain regions in PTSD as compared to PTSD+DS.

Individuals with PTSD+DS as compared to PTSD exhibited significantly stronger rsFC of the PPN with a cluster encompassing the right amygdala and the parahippocampal gyrus (*p*_FWE_ = .005), and a cluster encompassing the left anterior cingulate and the ventromedial prefrontal cortex (*p*_FWE_ = .011) (Table 2; Figure 2(b)).

### Relationship Between PPN rsFC and Clinical Characteristics

#### PTSD

In individuals with PTSD, we did not observe a significant association between rsFC of the PPN and PTSD symptom severity, childhood traumatization, depressive symptomatology, and state derealization/depersonalization.

#### PTSD+DS

**Figure 3.**
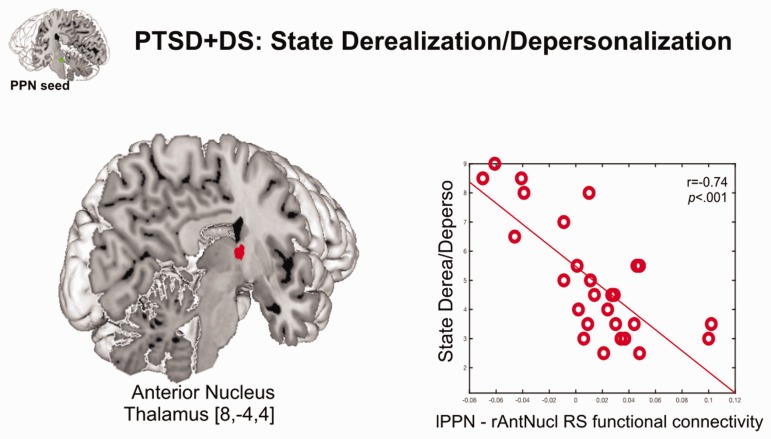
Negative correlation of state depersonalization/derealization with resting state functional connectivity (rsFC) between the left pedunculopontine nucleus and the right thalamus (anterior nucleus) in PTSD+DS. ROI approach: RsFC results are reported at a local significance threshold of *p* < .0125, FWE corrected (additionally adjusted for number of ROIs); only voxels surviving the latter threshold are displayed (binary, MNI space). Correlation with rsFC is displayed in red. The seed region (PPN) is displayed in green. The scatterplot represents the relationship between the extracted beta weights of the rsFC of the lPPN with the right anterior nucleus of the thalamus and state depersonalization/derealization. Derea: derealization; Deperso: depersonalization; lPPN: left pedunculopontine nucleus; rAntNucl: right anterior thalamic nucleus; RS: resting state; PTSD+DS: dissociative subtype of post-traumatic stress disorder; [x, y, z] = [x coordinate, y coordinate, z coordinate].

In individuals with PTSD+DS, higher PTSD symptom severity was related to reduced rsFC of the left PPN with the right caudate (*p*_FWE_ = .009). In addition, in individuals with PTSD+DS, increased state derealization/depersonalization was associated with reduced rsFC of the left PPN with the right anterior nucleus of the thalamus (*p*_FWE_ = .007) (Table 3; Figure 3). We did not observe a significant relationship between depressive symptomatology or childhood traumatization and rsFC of the PPN and any other brain region in individuals with PTSD+DS.

**Table 3.** Relationship between resting state functional connectivity of the left pedunculopontine nuclei and clinical characteristics in PTSD+DS individuals.

	PTSD+DS	L/R		Brain region		k	Z	*p*_FWEcorr_	*p*_uncorr_	Peak MNI Coordinate		
										x	y	z
LPPN	PTSD symptom severity	R	↓	Caudate	**	79	4.38	.009	<.001	4	0	4
	State derealization/depersonalization	R	↓	Thalamus (anterior nucleus)	**	124	4.45	.007	<.001	8	−4	4

ROI approach: rsFC results are reported at a local significance threshold of **p < .0125 (i.e., adjusted for number of ROIs), FWE corrected. PTSD: post-traumatic stress disorder; PTSD+DS: PTSD with the dissociative subtype; L: left; R: right; n.s.: no significant difference; k: cluster size; *p*_uncorr_: *p*-value, uncorrected for multiple comparisons; *p*_FWEcorr_: *p-*value, corrected for multiple comparisons (FWE); ↓: negative correlation; FWE: family-wise error; MNI: Montreal Neurological Institute; LPPN: left peduncolopontine nuclei.

## Discussion

The current investigation aimed to delineate patterns of rsFC with a main component of the RAS, the PPN, a brain region involved in general arousal and innate reflexive responding.^75^ Critically, as compared to both controls and PTSD, individuals with PTSD+DS showed increased rsFC of the PPN with a cluster encompassing the amygdala and the parahippocampal gyrus and a cluster encompassing the anterior cingulate and the ventromedial prefrontal cortex, brain regions involved in innate threat processing and arousal.^18,19,91–93,95–97^ Among individuals with PTSD, we did not observe differences in PPN rsFC when compared to controls. Interestingly, in individuals with PTSD+DS, increased state derealization/depersonalization was associated with decreased rsFC between the PPN and the anterior nucleus of the thalamus, a pattern that may contribute, in part, to reduced RAS-thalamo-cortical transmission of interoceptive and exteroceptive signals during states of derealization and depersonalization.^98^

### PPN rsFC With Brain Regions Involved in Innate Threat and Arousal Processing

Deep-layer neuronal circuits that control innate and learned reflexive responses as well as arousal^99–101^ are becoming increasingly important in the neurobiological conceptualization of PTSD.^1,2,14–16,18,19,65–67,99–103^ Crucially, these fundamental neuronal circuits determine the general arousal state of the organism, laying the foundation for reflexive actions.^26^ The present investigation provides novel evidence that as compared to both control subjects and individuals with PTSD, participants with PTSD+DS showed stronger rsFC of the PPN with the amygdala, the parahippocampal gyrus, the anterior cingulate cortex, and the ventromedial prefrontal cortex.^91–93,95–97^ Taken together, these results reveal increased PPN rsFC to subcortical and cortical brain regions in individuals with PTSD+DS.

Interestingly, the subcortical and the cortical brain regions described above are involved in the innate alarm system, a network of brain regions facilitating “fast-tracked” activation of alerting and defense responses.^91–93,95^ Although there is direct communication between deep-layer brain structures of the innate alarm system and cortical brain regions, deep-layer brain regions of the innate alarm system are critical in initiating subliminal, fast responses (i.e., instinctual defense responses via the adaption of physiological arousal), which in turn activate higher level cortical brain regions.^91–93,95–97^ Here, we provide the first evidence of increased resting state connectivity between subcortical and cortical components of the innate alarm system and the RAS in individuals with PTSD+DS. This pattern of enhanced connectivity with cortical brain regions may be in keeping with previous observations indicative of cortical top-down regulation of deeper-layer innate alarm system and RAS brain structures in PTSD+DS.^13–15^ In addition, enhanced connectivity with the amygdala and the parahippocampal gyrus could indicate modulation of deeper-layer innate alarm regions in PTSD+DS.^62^ Further research examining the relation between brainstem, limbic, and cortical neural circuits at rest and in response to symptom provocation is needed urgently.

We did not detect patterns of altered PPN rsFC with any other brain region in PTSD as compared to PTSD+DS and controls. The PPN aids greatly in the integration of incoming sensory information,^41,104,105^ a process highly relevant to the clinical model of PTSD+DS, where reduced awareness of the environment and of bodily states characterizes individuals with the dissociative subtype of PTSD. Further research investigating the heterogeneous neurobiological processes underlying different subtypes of PTSD is needed.

### The RAS and Its Relation to Derealization and Depersonalization Experiences

In participants with PTSD+DS, heightened states of derealization/depersonalization were related to reduced rsFC between the PPN and the anterior nucleus of the thalamus. Thalamic nuclei, particularly the anterior, the medial dorsal nuclei, and the pulvinar nuclei, play a pivotal role in controlling intrinsic alertness,^106,107^ where intrinsic alertness is defined as a fundamental state of arousal in the absence of any external input. This individual level of intrinsic alertness thus determines readiness to react.^108–111^ Critically, states of derealization/depersonalization, that is, psychological defense strategy to trauma, when no physical escape is possible, involve reduced responsiveness to sensory stimuli and hence reduced behavioral action generation,^14^ while importantly, this has been associated previously with cortical-sensory deafferentation. Here, the inhibition of the thalamus is stated to restrict the excitation and hence, the somatosensory information transmission to higher order cortical brain structures.^14,98^ The present study provides evidence that thalamic engagement (i.e., anterior nucleus) is related to a key RAS brain structure (i.e., PPN).^112^ The latter may be critical to establish readiness to react,^71–73,75,106–110^ while importantly, this is found to be reduced during states of derealization/depersonalization in PTSD+DS.^14,19,113–122^ Hence, as reduced thalamic engagement has been reported repeatedly in PTSD, with most pronounced changes observed in PTSD+DS,^5,123–126^ the present investigation extends these findings by highlighting the importance of deep-layer neuronal circuitries in the functioning of higher brain structures involved in depersonalization.

### Limitations

There are several limitations to the current investigation. The present investigation was based on 3T fMRI data. As the brainstem comprises relatively small nuclei, future studies using high-resolution fMRI would allow for a more thorough investigation of these nuclei at an enhanced spatial resolution. Task-related studies triggering the RAS specifically would be helpful in gaining further insights into the temporal dynamics of this critical system and the contrasting neural signatures of PTSD and its dissociative subtype.

## Conclusion

The present investigation revealed distinct alterations in deep-layer reflexive responding and arousal-related neuronal circuitries in PTSD and its dissociative subtype during rest. Whereas both PTSD groups exhibited within-group rsFC of the PPN with brain regions implicated in innate threat processing and arousal, as compared to both the control and PTSD groups, only the PTSD+DS group exhibited stronger rsFC of the PPN with a cluster encompassing the amygdala and the parahippocampal gyrus and a cluster encompassing the anterior cingulate and the ventromedial prefrontal cortex. Taken together, these results highlight the central role of instinctual reflexive responding in PTSD+DS. Critically, in PTSD+DS, increased state derealization/depersonalization was related to reduced PPN and thalamus rsFC, likely reflecting reduced RAS-thalamo-cortical transmission of intero- and exteroceptive signals, thus limiting an individual's perception not only of the condition of one's body but also of one's self in relation to the environment. The latter may serve as an important mechanism underlying depersonalization/derealization. Finally, the present study highlights the necessity of incorporating fundamental brainstem circuitries, including the RAS, into studies seeking to identify the neurobiological underpinnings and clinical characteristics of PTSD and its dissociative subtype.[Fig fig1-2470547019873663]

## Supplemental Material

CSS873663 Supplemental Material - Supplemental material for Back to the Basics: Resting State Functional Connectivity of the Reticular Activating System in PTSD and its Dissociative Subtype Click here for additional data file. Supplemental material, CSS873663 Supplemental Material for Back to the Basics: Resting State Functional Connectivity of the Reticular Activating System in PTSD and its Dissociative Subtype by Janine Thome, Maria Densmore, Georgia Koppe, Braeden Terpou, Jean Théberge, Margaret C. McKinnon and Ruth A. Lanius in Chronic Stress
