# Supplementary material for: Back to the Basics: Resting State Functional Connectivity of the Reticular Activation System in PTSD and its Dissociative Subtype
Source: Chronic Stress (Thousand Oaks). 2019 Sep 27;3:2470547019873663. doi: 10.1177/2470547019873663 (PMC7219926; doi:10.1177/2470547019873663)
Supplement: CSS873663 Supplemental Material - Supplemental material for Back to the Basics: Resting State Functional Connectivity of the Reticular Activating System in PTSD and its Dissociative Subtype [file CSS873663_Supplemental_Material.pdf]

S1 Supplemental Methods

Sample Description: Exclusion criteria

The present sample represents a subgroup of a previously published larger sample (see (1-8). Here, we excluded five healthy control individuals to avoid differences in age across groups. In addition, two PTSD+DS, as well as two PTSD subjects were excluded as their brainstem had not been scanned completely (functional magnetic resonance imaging and anatomical magnetic resonance imaging, respectively). In general, participants were excluded if they had implants or metal that do not comply with 3T fMRI safety standards for research, a history of head injury with a loss of consciousness, significant untreated medical illness, a history of neurological disorders, history of any pervasive developmental disorders, pregnancy, and current use of any psychotropic medication within one month prior to study. PTSD individuals were further excluded if they reported a history of bipolar disorder, schizophrenia, or substance-use disorder prior to participation of the study.

Statistical analysis: Sample characteristics

Age, PTSD symptom severity (CAPS total score), severity of childhood traumatization (CTQ total score), trait dissociative experiences (MDI total score, MDI derealization subscale, MDI depersonalization subscale), as well as state dissociative experiences (RSDI derealization and depersonalization), and state anxiety (STAI-S) were compared using one-way analysis of variance (ANOVA; multivariate ANOVA has been applied to compare MDI subscales). Gender and comorbid diagnosis were contrasted using Kruskall-Wallis Test.

Statistical significance was set to $p<0.05$, and in case of significant effects, Bonferroni-corrected, post-hoc analyses were applied. All analyses were performed using SPSS (version 25; SPSS Inc., USA).

RS functional connectivity: Analysis approach and statistical thresholding of the partial-brain analysis

RsFC analyses to voxels within the brainstem/cerebellum only (SUIT-space analyses, no additional ROI approach) were thresholded as follows: Within-group analyses were tested at a local significance threshold of $p<0.05$ (voxel level), corrected for multiple comparisons (FWE). Between-group analyses were tested at a local significance threshold of $p<0.05$, uncorrected for multiple comparisons and results are reported that passed a threshold of $p<.005$, uncorrected for multiple comparisons.

Table S1. Resting state functional connectivity of the left pedunculopontine nuclei and brainstem and cerebellar regions within each group (one sample t-test, partial-brain level, i.e. SUIT space)

	L/R	Brain Region		k	Z	*p*FWE-corr	*p*uncorr	Peak MNI Coordinate		
								x	y	z
**controls**										
	L	Anterior Cerebellum I-IV	**	5	4.451	0.009	<.001	-2	-44	-19
**PTSD**										
	L	Peduncolopontine Nuclei/Locus Coeruleus	**	1258	Inf	<.001	<.001	-6	-32	-23
	R	Peduncolopontine Nuclei/Locus Coeruleus	**		7.390	<.001	<.001	8	-34	-21
	R	Superior Colliculi/midbrain RF	**		6.145	<.001	<.001	8	-32	-13
	R	Posterior Cerebellum VI	**	5	5.041	0.002	<.001	30	-52	-27
	L	Vermis Cerebellum IV-V	**	6	4.881	0.010	<.001	2	-50	-15
**PTSD+DS**										
	L	Locus Coeruleus	**	747	Inf	<.001	<.001	-4	-32	-21
	R	Anterior Cerebellum I-IV	**		6.130	<.001	<.001	20	-32	-25
	R	Peduncolopontine Nuclei	**		6.058	<.001	<.001	10	-32	-23

**p<0.05 (voxel-level), FWE corrected

Legend: PTSD = post-traumatic stress disorder; PTSD+DS = PTSD with the dissociative subtype; n.s.=no significant difference ; k = cluster size; p_{uncorr} = p-value, uncorrected for multiple comparisons; $p_{FWEcorr}$ = p-value, corrected for multiple comparisons (family wise error)

Table S2. Resting state functional connectivity of the right pedunculopontine nuclei and brainstem and cerebellar regions within each group (one sample t-test, partial-brain level, i.e. SUIT space)

	Brain Region		k	Z	*p*FWE-corr	*p*uncorr	Peak MNI Coordinate x	y	z
**controls**									
		n.s.							
**PTSD**									
R	Peduncolopontine Nuclei/Locus Coeruleus	**	1417	Inf	<.001	<.001	8	-32	-23
L	Peduncolopontine Nuclei/Locus Coeruleus	**		7.149	<.001	<.001	-10	-30	-21
L	Posterior Cerebellum VI	**		6.832	<.001	<.001	-12	-38	-23
R	Anterior Cerebellum V	**	6	5.085	0.001	<.001	8	-62	-21
R	Posterior Cerebellum VI	**	6	4.852	0.004	<.001	20	-58	-23
**PTSD+DS**									
R	Peduncolopontine Nuclei/Locus Coeruleus	**	465	Inf	<.001	<.001	8	-32	-21
L	Periaqueductal Gray	**		6.797	<.001	<.001	-2	-34	-21
R	Ventral Tegmental Area	**	23	5.063	0.001	<.001	2	-24	-15
L	Substantia Nigra	**		4.877	0.003	<.001	-10	-16	-9
R	Substantia Nigra	**		4.415	0.022	<.001	-4	-18	-17
R	Anterior Cerebellum V	**	6	4.850	0.003	<.001	28	-42	-25
R	Anterior Cerebellum I-IV	**	16	4.789	0.005	<.001	0	-46	-17
R	Anterior Cerebellum I-IV	**		4.535	0.014	<.001	0	-38	-13

**$p<0.05$ (voxel-level), FWE corrected

Legend: PTSD = post-traumatic stress disorder; PTSD+DS = PTSD with the dissociative subtype; n.s.=no significant difference ; k = cluster size; p_{uncorr} = p-value, uncorrected for multiple comparisons; $p_{FWEcorr}$ = p-value, corrected for multiple comparisons (family wise error)

Table S3. Between-group comparisons of resting state functional connectivity of the left and right pedunculopontine nuclei and brainstem, as well as cerebellar regions (partial-brain level, i.e. SUIT space)

	L/R	Brain Region	k	Z	*p*FWE-corr	*p*uncorr	Peak MNI Coordinate x	y	z
**Main Effect of Group**									
		n.s.							
**Interaction Effect**									
		n.s.							
**Between-Group Effects**									
controls > PTSD		n.s.							
controls > PTSD+DS		n.s.							
PTSD > controls		n.s.							
PTSD > PTSD+DS		n.s.							
PTSD+DS>controls	R	Anterior Cerebellum I-IV	9	3.075	0.227	0.001	8	-48	-27
	L	Anterior Cerebellum I-IV	9	2.931	0.313	0.002	-6	-52	-21
PTSD+DS>controls	L	Anterior Cerebellum V	7	3.452	0.083	<.001	-8	-54	-11

**$p_{\text{uncorrected}}$<0.005 (voxel-level), k>5.

<u>Legend:</u> PTSD = post-traumatic stress disorder; PTSD+DS = PTSD with the dissociative subtype; n.s.=no significant difference; k = cluster size; p_{uncorr} = p-value, uncorrected for multiple comparisons; $p_{FWEcorr}$ = p-value, corrected for multiple comparisons (FWE)

Table S4. ROI resting state functional connectivity of the left pedunculopontine nuclei within each group (one sample t-test; whole-brain level)

							Peak MNI Coordinate		
**L/R**	**Brain Region**		**k**	**Z**	$p_{FWE\text{-}corr}$	p_{uncorr}	**x**	**y**	**z**
**CONTROLS**									
		n.s.							
**PTSD**									
L	Thalamus (anterior, lateral dorsal, pulvinar)	**	231	4.24	0.003	<.001	-6	-16	14
R	Thalamus (anterior, lateral dorsal, pulvinar)	**	136	3.79	0.011	<.001	16	-28	14
R	Ventromedial Prefrontal Cortex/Anterior Cingulate Cortex	**	539	4.23	0.004	<.001	14	60	-2
**PTSD+DS**									
L	Amygdala/Parahippocampal Gyrus	**	306	4.70	<.001	<.001	-20	2	-16
R	Ventromedial Prefrontal Cortex/Anterior Cingulate Cortex	**	384	4.09	0.005	<.001	16	-14	18
R	Ventromedial Prefrontal Cortex/Anterior Cingulate Cortex	**	610	3.88	0.009	<.001	12	52	-8
R	Amygdala/Parahippocampal Gyrus	**	152	3.92	0.003	<.001	28	-4	-14

ROI approach: RS functional connectivity results are reported at a local significance threshold of **p<0.0125 (i.e., adjusted for number of ROIs), FWE corrected

Legend: PTSD = post-traumatic stress disorder; PTSD+DS = PTSD with the dissociative subtype; n.s.=no significant difference ; k = cluster size; p_{uncorr} *= p-value, uncorrected for multiple comparisons;* $p_{FWEcorr}$ *= p-value, corrected for multiple comparisons (family wise error)*

Table S5. ROI Resting state functional connectivity of the right pedunculopontine nuclei within each group (one sample t-test, whole-brain level)

L/R	Brain Region		k	Z	$p_{FWE\text{-}corr}$	p_{uncorr}	Peak MNI Coordinate x	y	z
**CONTROLS**									
R	Thalamus (anterior nucleus)	**	91	4.57	0.001	<.001	8	0	6
**PTSD**									
R	Thalamus (anterior, medial dorsal, lateral dorsal, pulvinar)	**	716	5.97	<.001	<.001	12	-2	12
L	Amygdala/Parahippocampal Gyrus	**	82	4.84	<.001	<.001	-26	-2	-12
L	Thalamus (anterior, medial dorsal, lateral dorsal, pulvinar)	**	220	4.51	0.001	<.001	-12	-12	18
R	Ventromedial Prefrontal Cortex	**	486	4.15	0.005	<.001	2	64	-10
**PTSD+DS**									
R	Thalamus (anterior, lateral dorsal, pulvinar)	**	292	4.00	0.006	<.001	16	-12	18
L	Amygdala/Parahippocampal Gyrus	**	116	3.65	0.006	<.001	-28	-4	-12
L	Thalamus (medial dorsal nucleus, pulvinar)	**	332	3.93	0.009	<.001	-6	-16	10
R	Amygdala/Parahippocampal Gyrus	**	207	3.57	0.009	<.001	24	0	-12
L	Ventromedial Prefrontal Cortex	**	503	3.75	0.012	<.001	-2	48	-10

ROI approach: RS functional connectivity results are reported at a local significance threshold of **p<0.0125 (i.e., adjusted for number of ROIs), FWE corrected

Legend: PTSD = post-traumatic stress disorder; PTSD+DS = PTSD with the dissociative subtype; n.s.=no significant difference; k = cluster size; p_{uncorr} *= p-value, uncorrected for multiple comparisons;* $p_{FWEcorr}$ *= p-value, corrected for multiple comparisons (family wise error)*

Table S6. Between-group comparison of resting state functional connectivity of the pedunculopontine nuclei (whole-brain level)

	L/R	Brain Region	k	Z	*p*FWE-corr	*p*uncorr	Peak MNI Coordinate x	y	z
**Main Effect of Group**									
	R	Fusiform Gyrus	16	4.276	0.209	<.001	28	-78	-8
	R	Superior Frontal Gyrus	15	4.141	0.324	<.001	14	34	44
	R	Superior Occipital Gyrus	14	3.750	0.789	<.001	22	-92	6
	L	Calcarine	18	3.744	0.795	<.001	-8	-88	-2
	R	Caudate	20	3.671	0.866	<.001	16	16	-4
	R	Anterior Cingulate Cortex	12	3.619	0.907	<.001	20	40	14
	L	Orbitofrontal Gyrus	13	3.615	0.910	<.001	-2	50	-12
	R	Middle Occipital Gyrus	15	3.605	0.917	<.001	30	-80	8
**Main Effect of Hemisphere**									
		n.s.							
**Interaction Effect of Group x Hemisphere**									
		n.s.							
**Between-Group Comparison**									
controls > PTSD	R	Supplementary Motor Area	10	3.442	0.956	<.001	14	-16	54
controls > PTSD+DS		n.s.							
PTSD > controls	R	Anterior Cingulate Cortex	46	4195	0.227	<.001	20	40	14
	R	Caudate Nucleus	20	3821	0.619	<.001	34	-40	10
	R	Superior Frontal Gyrus	21	3657	0.805	<.001	22	58	4
	R	Caudate Nucleus	20	3559	0.890	<.001	18	14	8
	R	Caudate Nucleus	15	3485	0.936	<.001	16	10	20
	R	Middle Frontal Gyrus	17	3482	0.938	<.001	44	6	38
PTSD>PTSD+DS	R	Medial Superior Frontal Gyrus	27	4466	0.086	<.001	12	34	44
	R	Middle Frontal Gyrus	15	3902	0.520	<.001	24	48	32
	R	Middle Frontal Gyrus	14	3823	0.616	<.001	30	28	30
	R	Middle Frontal Gyrus	20	3719	0.739	<.001	26	14	56
	R	Medial Superior Frontal Gyrus	21	3560	0.889	<.001	8	42	48
	R	Middle Frontal Gyrus		3488	0.935	<.001	18	42	46

PTSD+DS>controls	R	Caudate	89	4243	0.194	<.001	16	16	-4
	R	Lateral Globus Pallidus		3341	0.984	<.001	16	6	-12
	L	Orbitofrontal Gyrus	79	4145	0.267	<.001	-2	50	-12
	L	Anterior Cingulate Cortex		3416	0.965	<.001	-4	44	-2
	R	Superior Occipital Gyrus	65	3825	0.614	<.001	24	-86	8
	R	Interior Occipital Gyrus		3557	0.891	<.001	32	-84	-2
	R	Fusiform Gyrus	18	3774	0.675	<.001	32	-62	-14
	R	Fusiform Gyrus	22	3707	0.752	<.001	28	-78	-8
	R	Calcarine	31	3691	0.770	<.001	2	-78	6
	L	Calcarine		3198	0.998	.001	-6	-72	8
	L	Cuneus	10	3562	0.887	<.001	-6	-98	6
	R	Fusiform Gyrus	14	3485	0.936	<.001	34	-38	-18
	R	Superior Frontal Gyrus	12	3424	0.962	<.001	18	54	8
PTSD+DS>PTSD	R	Fusiform Gyrus	24	4534	0.066		28	-78	-8
	R	Superior Occipital Gyrus	74	4315	0.151		22	-92	8
	R	Lingual Gyrus		3680	0.782		24	-90	-2
	L	Calcarine	58	4211	0.216		-8	-88	-2
	R	Middle Occiputal Gyrus	35	4138	0.272		30	-80	8
	L	Interior Temporal Gyrus	19	4049	0.355		-48	-58	-14
	L	Superior Temporal Gyrus	21	3854	0.577		-46	-26	2
	R	Superior Temporal Gyrus	11	3726	0.731		46	-32	2
	R	Amygdala	13	3713	0.745		28	-6	-14
	R	Lentiform Nucleus		3131	0.999		34	0	-12
	L	Orbitofrontal Gyrus	10	3710	0.749		-12	54	-2
	L	Middle Occipital Gyrus	24	3698	0.762		-36	-76	8
	R	Inferior Occipital Gyrus	20	3697	0.763		38	-66	-6
	R	Fusiform Gyrus		3522	0.915		32	-64	-14
	R	Calcarine	11	3645	0.817		18	-84	2
	R	Lingual Gyrus	13	3602	0.856		36	-76	-4
	R	Calcarine	13	3594	0.863		4	-78	6
	L	Lingual Gyrus	12	3563	0.887		-16	-80	-8
	L	Orbitofrontal Gyrus	10	3504	0.926		-6	36	-14

Whole-brain RS functional connectivity results are reported at a threshold of *p*<0.001 (voxel-level), k>10, uncorrected for multiple comparisons

<u>*Legend:*</u> *PTSD = post-traumatic stress disorder; PTSD+DS = PTSD with the dissociative subtype; n.s.=no significant difference; k = cluster size;* p_{uncorr} *= p-value, uncorrected for multiple comparisons;* $p_{FWEcorr}$ *= p-value, corrected for multiple comparisons (family-wise error)*

S3 References

1. Harricharan S, Rabellino D, Frewen PA, Densmore M, Theberge J, McKinnon MC, et al. fMRI functional connectivity of the periaqueductal gray in PTSD and its dissociative subtype. Brain and behavior. 2016;6(12):e00579.

2. Nicholson AA, Sapru I, Densmore M, Frewen PA, Neufeld RW, Theberge J, et al. Unique insula subregion resting-state functional connectivity with amygdala complexes in posttraumatic stress disorder and its dissociative subtype. Psychiatry Res Neuroimaging. 2016;250:61-72.

3. Harricharan S, Nicholson AA, Densmore M, Theberge J, McKinnon MC, Neufeld RWJ, et al. Sensory overload and imbalance: Resting-state vestibular connectivity in PTSD and its dissociative subtype. Neuropsychologia. 2017;106:169-78.

4. Thome J, Densmore M, Frewen PA, McKinnon MC, Théberge J, Nicholson AA, et al. Desynchronization of autonomic response and central autonomic network connectivity in posttraumatic stress disorder. Human brain mapping. 2017;38(1):27-40.

5. Nicholson AA, Densmore M, McKinnon MC, Neufeld RWJ, Frewen PA, Theberge J, et al. Machine learning multivariate pattern analysis predicts classification of posttraumatic stress disorder and its dissociative subtype: a multimodal neuroimaging approach. Psychological medicine. 2018:1-11.

6. Rabellino D, Densmore M, Harricharan S, Jean T, McKinnon MC, Lanius RA. Resting-state functional connectivity of the bed nucleus of the stria terminalis in post-traumatic stress disorder and its dissociative subtype. Human brain mapping. 2018;39(3):1367-79.

7. Rabellino D, Densmore M, Theberge J, McKinnon MC, Lanius RA. The cerebellum after trauma: Resting-state functional connectivity of the cerebellum in posttraumatic stress disorder and its dissociative subtype. Human brain mapping. 2018;39(8):3354-74.

8. Terpou BA, Densmore M, Theberge J, Frewen P, McKinnon MC, Lanius RA. Resting-state pulvinar-posterior parietal decoupling in PTSD and its dissociative subtype. Human brain mapping. 2018.
